# Deferoxamine, deferasirox, and deferiprone triple iron chelator combination therapy for transfusion-dependent β-thalassaemia with very high iron overload: a randomised clinical trial

**DOI:** 10.1016/j.lansea.2024.100495

**Published:** 2024-10-15

**Authors:** Anuja Premawardhena, Sakuni Wanasinghe, Chamodi Perera, Muditha Nayana Wijethilaka, R.H.M.G. Rajakaruna, R.A.N.K.K. Samarasinghe, Senani Williams, Sachith Mettananda

**Affiliations:** a Colombo North Teaching Hospital, Ragama, 11010, Sri Lanka; b Department of Medicine, Faculty of Medicine, University of Kelaniya, Thalagolla Road, Ragama, 11010, Sri Lanka; c Department of Paediatrics, Faculty of Medicine, University of Kelaniya, Thalagolla Road, Ragama, 11010, Sri Lanka; d Department of Radiology, Lady Ridgeway Children's Hospital, Colombo, 00700, Sri Lanka; e Department of Pathology, Faculty of Medicine, University of Kelaniya, Thalagolla Road, Ragama, 11010, Sri Lanka

**Keywords:** Thalassaemia, Deferoxamine, Deferasirox, Deferiprone, Iron overload, Transfusion-dependent anaemia, Iron chelator

## Abstract

**Background:**

Many patients with β-thalassaemia die prematurely due to iron overload. In this study, we aim to evaluate the efficacy and safety of the triple combination of deferoxamine, deferasirox and deferiprone on iron chelation in patients with transfusion-dependent β-thalassaemia with very high iron overload.

**Methods:**

This open-label, randomised, controlled clinical trial was conducted at Colombo North Teaching Hospital, Sri Lanka. Transfusion-dependent β-thalassaemia patients with ferritin >3500 ng/mL were randomised 2:1 into intervention (deferoxamine, deferasirox and deferiprone) and control (deferoxamine and deferasirox) arms. Reduction in serum ferritin after six months was the primary outcome measure. Reduction in liver iron content, improvement in cardiac T2∗, and adverse effects were secondary outcome measures.

**Findings:**

Twenty-three patients (intervention-15, control-8) were recruited. 92% and 62% in the intervention and control arms showed a reduction in ferritin, respectively. The mean reduction of ferritin was significantly higher in intervention (−1094 ± 907 ng/mL) compared to control (+82 ± 1588 ng/mL) arm (p = 0.042). There was no statistically significant difference in the liver iron content in two arms. In the intervention arm, 67% improved cardiac T2∗ (mean change +6.72 ± 9.63 ms) compared to 20% in the control arm (mean change −3.00 ± 8.24 ms). Five patients discontinued deferiprone due to arthralgia, which resolved completely after stopping the drug.

**Interpretation:**

Triple combination therapy with deferoxamine, deferasirox and deferiprone is more efficacious in reducing iron burden measured by serum ferritin and showed a positive trend in reducing myocardial iron content in patients with transfusion-dependent β-thalassaemia with very high iron overload. Deferiprone has the disturbing side effect of reversible but severe arthropathy.

**Funding:**

None.

**Research in context**

**Evidence before this study**

Despite advances in blood transfusion and supportive management, patients with transfusion-dependent β-thalassaemia die prematurely due to iron overload-related complications. Deferoxamine, deferasirox, and deferiprone are the only available iron chelators at present. Combining two iron chelators is advocated in situations of high iron load despite using the maximum dose of a single drug. Nonetheless, some patients with transfusion-dependent β-thalassaemia continue to have high iron overload despite being on the maximum doses of two iron chelators. The treating clinicians have no options or alternatives when this scenario arises, as evidence for the simultaneous use of all three iron chelators is lacking. A PubMed search on 20 June 2024, using search terms ‘thalassaemia’, ‘iron chelators’, and ‘combination’, did not reveal a single randomised clinical trial assessing the efficacy and safety of the triple combination of deferoxamine, deferasirox and deferiprone on iron chelation in patients with transfusion-dependent β-thalassaemia.

**Added value of this study**

In this randomised open-label controlled clinical trial, we found that triple combination therapy with deferoxamine, deferasirox and deferiprone is more efficacious in reducing the overall iron burden as measured by serum ferritin and shows a positive trend in reducing myocardial iron content in patients with transfusion-dependent β-thalassaemia with very high iron overload. However, we did not observe a difference in the liver iron content. Deferiprone has the disturbing side effect of reversible but severe arthropathy.

**Implications of all the available evidence**

Our findings support the use of combination of deferoxamine, deferasirox, and deferiprone to chelate iron in transfusion-dependent β-thalassaemia patients with very high iron overload if the adverse effect profile is tolerable.

## Introduction

β-Thalassaemia is a genetic disorder of haemoglobin synthesis characterised by severe anaemia from early infancy.^1^ The autosomal recessive mutations of the β-globin gene lead to impaired synthesis of β-globin and adult haemoglobin, resulting in premature destruction of red blood cells.^2^ Patients with severe forms of β-thalassaemia are transfusion-dependent for life. Owing to the increased availability of safe blood for transfusion, β-thalassaemia has transformed from a fatal disease in childhood to a chronic disease with disability.^3^ Still, many patients with β-thalassaemia, those who live in developing countries in particular, die prematurely due to complications of blood transfusions and iron overload.^4^

Iron overload in β-thalassaemia is caused by two main mechanisms. Firstly, a single unit of blood contains approximately 200 mg of iron; thus, regular 2–5 weekly transfusions received by patients with β-thalassaemia lead to massive iron overload.^5^ Secondly, gastrointestinal absorption of iron is increased in patients with β-thalassaemia due to the suppression of hepcidin.^6^ In the presence of high plasma iron, transferrin is saturated, resulting in large quantities of non-transferrin-bound free iron, which are taken up selectively by hepatocytes and myocardial and endocrine cells, leading to tissue damage, organ dysfunction and failure.^7^

The only effective remedy for iron overload is therapeutic iron chelation. Three iron chelators–deferoxamine, deferasirox, and deferiprone–are available to chelate excess iron. They are recommended to be used as monotherapy in patients with β-thalassaemia to reduce serum ferritin to levels below 1000 ng/mL.^8^ The combination of two iron chelators is advocated in situations of high iron load despite using the maximum dose of a single drug. Deferoxamine and deferasirox are the most widely used and efficacious iron chelator combination.^9^ Nonetheless, some patients with transfusion-dependent β-thalassaemia continue to have high iron overload despite being on the maximum doses of deferoxamine and deferasirox.^10^ The treating clinicians have no options or alternatives when this scenario arises. Such patients continue to accumulate iron, develop iron overload-related complications and die prematurely during the third or fourth decades of life.^11^

The development of new iron chelator medications is a lower priority for the pharmaceutical industry due to challenges in development and the relatively smaller profit margins. Hence, the clinicians are left with only three medications for chelate excess iron.^12^ Thus, the only plausible approach for patients with iron overload, despite being on maximum doses of two iron chelators, is to combine all three iron chelators simultaneously. However, none of the guidelines recommend using all three medications together, and no clinical trial has evaluated the same. Therefore, in this study, we aim to evaluate the efficacy and safety of the triple combination of deferoxamine, deferasirox and deferiprone over the dual combination of deferoxamine and deferasirox on iron chelation in patients with transfusion-dependent β-thalassaemia with very high iron overload.

## Methods

### Study design

This open-label randomised controlled phase 2/3 clinical trial was conducted at the Thalassaemia Centre of the Colombo North Teaching Hospital, Ragama, Sri Lanka, from 01 June 2023 to 31 March 2024. The Colombo North Teaching Hospital is the second-largest thalassaemia centre and the only dedicated adolescent and adult thalassaemia centre in Sri Lanka, where adolescents and adults with thalassaemia from all parts of Sri Lanka are referred for treatment. The ethical approval for the trial was obtained from the Ethics Committee of the Faculty of Medicine, University of Kelaniya, Sri Lanka (P/06/02/2023) and the trial was registered in the Sri Lanka Clinical Trials Registry (SLCTR/2023/010) on 19 April 2023. The complete study protocol was published in BMJ Open (https://doi.org/10.1136/bmjopen-2023-077342).

### Participants

Patients with transfusion-dependent β-thalassemia with very high iron overload despite being on combination therapy with deferoxamine and deferasirox were eligible to participate in the trial. Transfusion dependency was defined as requiring at least eight blood transfusions (each with a minimum volume of 20 mL/kg) during the preceding 12 months, and very high iron overload was defined as having serum ferritin >3500 ng/mL in at least two measurements done over the preceding three months. The diagnosis of β-thalassaemia was done based on the results of the haemoglobin high-performance liquid chromatography (HPLC) done at the time of diagnosis; β-thalassaemia major was defined as haemoglobin F >90% with parental HPLC showing β-thalassaemia trait in patients presenting between 3 and 24 months and haemoglobin E β-thalassaemia was defined as haemoglobin F >50% and haemoglobin E >30%. Patients with age less than 12 years, pregnancy or lactation, active hepatitis B or C infection, history of severe adverse reactions to iron chelators, contraindications for any iron chelator or Magnetic Resonance Image (MRI) scanning, and those who were started on regular transfusions for a pre-determined period were excluded.

All eligible patients attending the study centre were recruited after obtaining informed written consent from the patient or from parent and patient (if aged between 12 and 17 years). The sample size was calculated to detect a 500 ng/mL difference in mean serum ferritin with an 80% power and 0.05 type I error for a 2:1 allocation ratio, assuming a population standard deviation of 400. The 2:1 allocation was done to make the study more attractive to participants and to gather additional safety information about the triple combination treatment. The minimum sample size required for intervention and control arms was twelve and six, respectively.

### Randomisation

Recruited participants were randomised into two groups using an online randomisation tool for an allocation ratio of 2:1 to intervention and control arms. The intervention arm received the combination of subcutaneous deferoxamine (Demeferidone, Demo SA, Greece) 25–40 mg/kg/day at least 5–7 days per week, oral deferasirox (Ciosyn, Celogen Lanka, Sri Lanka) 40 mg/kg/day, and oral deferiprone (Feripro, Highnoon Laboratory, Pakistan) 75 mg/kg/day for six months (triple therapy). The control arm received the combination of subcutaneous deferoxamine (Demeferidone, Demo SA, Greece) 25–40 mg/kg/day at least 5–7 days per week and oral deferasirox (Ciosyn, Celogen Lanka, Sri Lanka) 40 mg/kg/day for six months (dual therapy) (Supplementary Table S1).

### Procedures

Information on sociodemographic and clinical characteristics was gathered at the enrolment by interviewing patients and parents and perusing patient records. Patients were reviewed monthly during treatment to assess the adverse effects of trial medication, full blood count, alanine transaminases, aspartate transaminases and serum creatinine. Serum ferritin was measured using two-site immune-enzumometric assay performed by Tosoh AIA-360 analyser before commencing treatment and at three months and six months, whereas T2∗ MRI of the liver and heart were done using Siemens Magnetom Essenza 1.5 T MRI machine at recruitment and the completion of 6 months. Other standard treatments, including blood transfusion and evaluation of transfusion and iron overload-related complications, were continued per international guidelines.

### Outcomes

Reduction in iron overload, as measured by a reduction in serum ferritin, was the primary outcome measure. Reduction in liver iron content (LIC) measured by T2∗ MRI of the liver, improvement in myocardial iron content as measured by cardiac T2∗ value, and the frequency of adverse effects of trial medication were secondary outcome measures.

### Statistical analysis

The data was analysed using IBM SPSS Statistics 29.0 by intension-to-treat analysis. Continuous variables were presented as mean and standard deviations, and categorical variables were presented as frequency and percentages. The chi-square test and student's t-test were used in the analysis.

### Role of funding source

This clinical trial did not receive any specific funding.

## Results

We recruited 23 patients for the study. Fifteen were randomised to the intervention arm (triple therapy), while eight were randomised to the control arm (dual therapy).

### Characteristics of the study population

The sociodemographic features, clinical characteristics, and baseline laboratory parameters were similar in the intervention and control arms (Table 1).

**Table 1:** Socio-demographic and clinical characteristics of patients in intervention and control arms.

Characteristic	Intervention arm (n = 15)	Control arm (n = 8)
**Socio-demographic features**		
Sex		
Males [n (%)]	6 (40%)	5 (62.5%)
Females [n (%)]	9 (60%)	3 (37.5%)
Age, years [mean (±SD)]	24.7 (±6.0)	24.5 (±6.1)
**Clinical characteristics at recruitment**		
Diagnosis		
β-thalassaemia major [n (%)]	12 (80%)	8 (100%)
Haemoglobin E β-thalassaemia [n (%)]	3 (20%)	0 (0)
Splenectomised [n (%)]	6 (40%)	3 (37.5%)
Complications		
Diabetes [n (%)]	6 (40%)	2 (25%)
Hypothyroidism [n (%)]	3 (20%)	4 (50%)
Hypoparathyroidism [n (%)]	2 (13.3%)	6 (75.0%)
Delayed puberty [n (%)]	5 (33.3%)	7 (87.5%)
Vitamin D deficiency [n (%)]	10 (66.7%)	7 (87.5%)
Height, cm [mean (±SD)]	153.8 (±12.7)	149.6 (±10.8)
BMI, kg/m^2^ [mean (±SD)]	21.4 (±4.3)	20.1 (±4.2)
Average pre-transfusion haemoglobin in past 12 months, g/dL [mean (±SD)]	8.4 (±0.7)	8.5 (±0.5)
Total number of blood units received so far [mean (±SD)]	472 (±194)	620 (±251)
Lifetime highest serum ferritin, ng/mL [mean (±SD)]	10,806 (±4071)	10,586 (±2762)
Most recent serum ferritin before recruitment, ng/dL [mean (±SD)]	6012 (±2090)	5616 (±2300)
**Baseline laboratory parameters**		
Pre-transfusion haemoglobin, g/dL [mean (±SD)]	8.9 (±1.7)	9.1 (±0.9)
Total white cell count, x10^9^/L [mean (±SD)]	11.8 (±7.6)	10.9 (±4.9)
Platelet count, x10^9^/L [mean (±SD)]	252 (±149)	285 (±125)
Serum ferritin, ng/mL [mean (±SD)]	5789 (±2410)	4200 (±1290)
Liver iron content, mg/g [mean (±SD)]^a^	3.44 (±2.45)	2.74 (±1.69)
Cardiac T2∗, ms [mean (±SD)]^a^	15.2 (±9.9)	24.8 (±22.6)
Alanine transaminase, IU/L [mean (±SD)]	86.3 (±42.5)	69.8 (±50.1)
Aspartate transaminase, IU/L [mean (±SD)]	79.9 (±42.3)	56.0 (21.6)
Serum creatinine, μmol/L [mean (±SD)]	56.4 (±48.8)	47.1 (±9.6)

a Only 20/23 patients (intervention-14/15, control-6/8) had pre-treatment MRI.

**Fig. 1:**
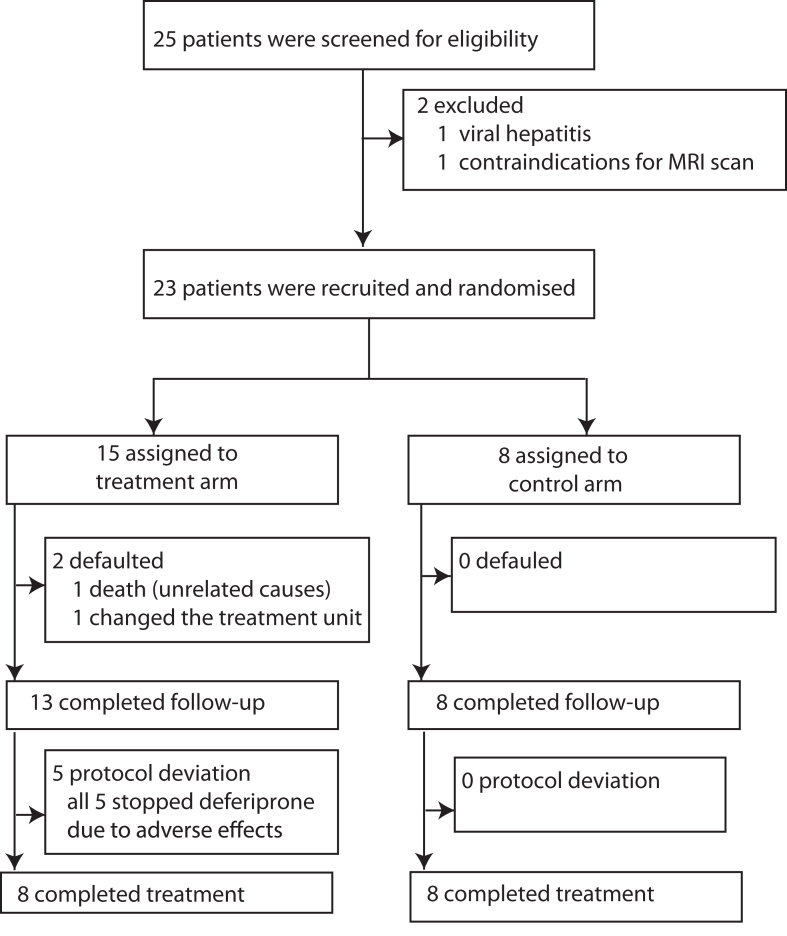
Trial profile.

All eight patients in the control arm completed the 6-month follow-up (Fig. 1). In the intervention arm, one patient died due to causes unrelated to trial medication, and one patient defaulted on the follow-up (Supplementary Table S2). Five patients deviated from treatment protocol and stopped trial mediation (deferiprone) at different time points due to the development of adverse effects (Supplementary Table S3).

### Effect on serum ferritin

We evaluated serum ferritin at the initiation and completion of three and six months of the trial (Table 2). 12/13 (92%) patients in the treatment arm showed a reduction in serum ferritin, while only 5/8 (62%) in the control arm showed reductions in serum ferritin compared to baseline after completion of 6-month treatment. The mean change of serum ferritin in the intervention arm was −1094 ± 907 ng/mL compared to +82 ± 1588 ng/mL in the control arm; this difference was statistically significant (p = 0.042). A comparable trend of higher reduction in serum ferritin was observed in the treatment arm compared to the control arm in the per-protocol analysis performed among the subset of participants who completed the trial per-protocol (Supplementary Table S4).

**Table 2:** Change of serum ferritin at 3 and 6 months compared to the baseline.

Number (%) showing reduction in serum ferritin compared to baseline	Intervention arm (n = 15)^a^	Control arm (n = 8)	OR (95% CI)	p-value
At the completion of 3 months	9 (64.3%)	6 (75%)	0.6 (0.1–4.1)	0.60
At the completion of 6 months	12 (92.3%)	5 (62.5%)	7.2 (0.6–87)	0.09

Number (%) showing >500 ng/mL reduction in serum ferritin compared to baseline	Intervention arm (n = 15)^a^	Control arm (n = 8)	OR (95% CI)	p-value
At the completion of 3 months	9 (64.3%)	3 (37.5%)	3.0 (0.5–18)	0.22
At the completion of 6 months	10 (76.9%)	4 (50.0%)	3.3 (0.5–22)	0.20

Change of serum ferritin from baseline (mean ± SD)	Intervention arm (n = 15)^a^	Control arm (n = 8)	Mean difference (95% CI)	p-value
At the completion of 3 months	−1576 (±2702)	−192 (±639)	−1384 (−3428 to 660)	0.173
At the completion of 6 months	−1094 (±907)	82 (±1588)	−1177 (−2309 to −44)	0.042

a Data of patients who defaulted the trial are not included; 1 defaulter at 3 months and 2 defaulters at 6 months.

### Effect on liver iron content

We performed the T2∗ MRI of the liver to evaluate the organ-specific iron overload at the initiation and the completion of 6 months of the trial. We did not observe a significant difference in the liver iron content between the intervention and control arms (Table 3). Comparable results were observed among the subset of participants who completed the trial per protocol (Supplementary Table S5).

**Table 3:** Change of liver iron content and cardiac T2∗ as measured by T2∗ MRI.^a^

Number (%) showing a decrease in liver iron content compared to baseline	Intervention arm (n = 12)	Control arm (n = 5)	OR (95% CI)	p-value
Any decrease in liver iron content	8 (66.7%)	3 (60.0%)	1.3 (0.1–11)	0.79
More than 10% decrease in the liver iron content	7 (58.3%)	3 (60.0%)	0.9 (0.1–7.8)	0.94

Change of liver iron content from baseline	Intervention arm (n = 12)	Control arm (n = 5)	Mean difference (95% CI)	p-value
Mean (±SD) change of liver iron content (mg/g)	−0.18 (±2.99)	0.75 (±3.26)	−0.9 (−4.4 to 2.5)	0.57

Number (%) showing increase in cardiac T2∗ compared to baseline	Intervention arm (n = 12)	Control arm (n = 5)	OR (95% CI)	p-value
Any increase in cardiac T2∗	8 (66.7%)	1 (20.0%)	8.0 (0.6–97)	0.07
More than 10% increase in cardiac T2∗	7 (58.3%)	1 (20.0%)	5.6 (0.5–66)	0.14

Change of cardiac T2∗ from baseline	Intervention arm (n = 12)	Control arm (n = 5)	Mean difference (95% CI)	p-value
Mean (±SD) change of cardiac T2∗ (ms)	6.72 (±9.63)	−3.00 (±8.24)	9.7 (−0.8 to 20)	0.06

a Only 17 participants (intervention-12; control-5) had pre- and post-treatment liver MRI due to limitation in facilities.

### Effect on cardiac iron

We performed cardiac T2∗ MRI to evaluate the myocardial iron content at the initiation and the completion of 6 months of the trial. 8/12 (67%) patients in the intervention arm showed increases in cardiac T2∗ after a 6-month trial period compared to 1/5 (20%) in the control arm (Table 3). The cardiac T2∗ increased by a mean of 6.72 ± 9.63 ms in the intervention arm, while cardiac T2∗ decreased by a mean of 3.00 ± 8.24 ms in the control arm, demonstrating superior cardiac iron chelation in the intervention arm. A comparable trend of improvement of cardiac T2∗ was observed in the intervention arm compared to the control arm in the per-protocol analysis performed among the subset of participants who completed the trial per-protocol (Supplementary Table S6).

### Adverse effects

Table 4 shows the adverse effects reported by trial participants. Eight participants in the intervention arm reported arthralgia, which was attributed to the trial medication deferiprone. Five of them discontinued deferiprone, and the arthralgia resolved completely after stopping the drug (Supplementary Table S7). Other adverse events reported more frequently in the intervention arm were abdominal discomfort, abdominal pain, increased appetite and fatigue. Abdominal pain and discomfort were transient and did not require medical treatment. All patients who experienced abdominal discomfort, abdominal pain, increased appetite and fatigue continued trial medication.

**Table 4:** Adverse effects.

Adverse effect	Intervention arm (n = 15)	Control arm (n = 8)
Constipation	0	0
Diarrhoea	0	0
Abdominal discomfort	4 (26.7%)	3 (37.5%)
Headache	0	0
Arthralgia	8 (53.3%)	0
Fever	0	0
Muscle pains	0	0
Skin rash	0	1 (12.5%)
Abdominal pain	3 (20%)	0
Agranulocytosis	0	0
Neutropenia	0	0
Increased appetite	2 (13.3%)	0
Fatigue	2 (13.3%)	0

Haematological parameters, hepatic transaminases and serum creatinine were not different between the intervention and control arms at 3-month and 6-month follow-up, showing the minimal effect of the triple combination treatment on haematological, hepatic and renal functions (Table 5).

**Table 5:** Haematological and biochemical parameters of intervention and control arms at 3-month and 6-month follow-up.

Parameter	Intervention arm (n = 13)	Control arm (n = 8)	p-value
**At 3-month follow-up**			
Pre-transfusion haemoglobin, g/dL [mean (±SD)]	8.7 (±0.8)	9.1 (±0.4)	p = 0.19
Total white cell count, x10^9^/L [mean (±SD)]	11.2 (±7.1)	11.3 (±5.0)	p = 0.97
Platelet count, x10^9/^L [mean (±SD)]	255 (±140)	294 (±133)	p = 0.53
Alanine transaminase, IU/L [mean (±SD)]	99 (±64)	65 (±45)	p = 0.20
Aspartate transaminase, IU/L [mean (±SD)]	63 (±23)	51 (±14)	p = 0.18
Serum creatinine, μmol/L [mean (±SD)]	51 (±14)	47 (±9)	p = 0.47
**At 6-month follow-up**			
Pre-transfusion haemoglobin, g/dL [mean (±SD)]	8.7 (±1.3)	8.9 (±1.4)	p = 0.68
Total white cell count, x10^9^/L [mean (±SD)]	12.9 (±9.9)	10.0 (±3.3)	p = 0.43
Platelet count, x10^9^/L [mean (±SD)]	270 (±147)	286 (±159)	p = 0.81
Alanine transaminase, IU/L [mean (±SD)]	86 (±64)	63 (±41)	p = 0.37
Aspartate transaminase, IU/L [mean (±SD)]	66 (±35)	57 (±23)	p = 0.51
Serum creatinine, μmol/L [mean (±SD)]	50 (±12)	50 (±12)	p = 0.99

## Discussion

Here, we present the results of the first-ever randomised controlled clinical trial evaluating the efficacy and safety of triple combination therapy of deferoxamine, deferasirox and deferiprone on iron chelation in transfusion-dependent β-thalassaemia. The study has many strengths. Firstly, the study includes a well-defined group of patients with transfusion-dependent β-thalassaemia with very high serum ferritin. Secondly, the study evaluated the efficacy of treatment comprehensively on multiple outcomes, including serum ferritin, T2∗ MRI of the liver and T2∗ MRI of the heart. Thirdly, the patients were carefully followed up to detect the development of adverse effects and complications related to trial medications.

The study revealed that the triple combination therapy with deferoxamine, deferasirox and deferiprone is superior to the dual combination therapy with deferoxamine and deferasirox in reducing the iron overload burden. Specifically, a significant reduction in serum ferritin was noted in patients receiving triple combination therapy. The chelation of myocardial iron assessed as cardiac T2∗ value was better in the triple combination group. The tendency to improve cardiac T2∗ even during the short period of the study was an important observation, even though only one patient with very low (<10 ms) cardiac T2∗ showed improvement to the safe limit of >20 ms.

The plausible explanation for the superior efficacy of the triple combination therapy is that three medications chelate iron via independent mechanisms. Deferoxamine binds iron in a 1:1 ratio to form a stable chelator–iron complex excreted via urine and faeces. Deferasirox binds to iron in a 2:1 chelator-to-iron ratio and is excreted via faeces. In contrast, three deferiprone molecules bind to a single iron molecule excreted in urine.^8^ Thus, it is reasonable to believe the combination of chelators has additive effects in chelating iron. As deferiprone is a low-cost iron chelator, its introduction to the combination iron chelator regimen would not significantly increase the cost of treatment.^13^

Despite beneficial effects on serum ferritin and myocardial iron, triple combination therapy did not improve the liver iron content compared to dual therapy. The lack of improvement of iron markers in the liver with the triple combination is difficult to explain. A longer trial would perhaps be needed to better comprehend this variable response.

Whether the ability to improve ferritin and cardiac T2∗ without altering liver iron content is due to unique features of the ‘third agent’ (i.e., deferiprone) or the sheer presence of three different chelators is difficult to explain. Deferiprone is thought to have special cardioprotective effects, even when used as monotherapy.^14^ However, it is premature to attribute the benefits seen in this data to a single drug alone. The addition of amlodipine to the iron chelator regimen has previously been shown to improve the cardiac T2∗ in patients with thalassaemia.^15^ Improved chelation of myocardial iron is linked to decreased incidence of iron-related cardiomyopathy and cardiac failure.^16^ Therefore, having the third chelator, in this case, deferiprone to the chelation regimen with deferoxamine and deferasirox, would potentially provide survival benefits to patients with β-thalassaemia by chelating myocardial iron and reducing iron overload-related complications.

The main drawback of the triple combination therapy observed in the study was the higher prevalence of adverse effects in the intervention group. Arthralgia was the most frequent complication and was severe enough for 5/15 (33%) patients to stop the drug. However, arthralgia was reversible and resolved completely within a few days after stopping deferiprone. Arthralgia is a well-recognised adverse effect of deferiprone.^17^ It has previously been reported that patients in South Asia are more prone to develop arthralgia related to deferiprone, which could be due to a genetic susceptibility in these ethnicities.^18^^,^^19^ In contrast, deferiprone is widely used as a monotherapy in Thailand and Mediterranean countries, where the occurrence of arthralgia is less.^17^^,^^20^ Thus, the increased frequency of adverse effects in the intervention arm could be a feature unique to Sri Lankan ethnicity, and the triple combination could have improved tolerability in other parts of the world. However, the presence of severe adverse effects in the intervention arm during the short trial period of 6 months raises concerns over the tolerability of triple combination therapy long-term.

One important limitation of our study is that it was an open-label study. We did not include a placebo due to the cost and technical difficulties. As the primary and secondary endpoints measuring the efficacy of the treatment were objective laboratory parameters, the bias created by perceived knowledge of the treatment arm would have had minimal effect on the efficacy assessment. However, the knowledge of the treatment arm could have created a bias in evaluating adverse effects, which were mostly subjective.

Another limitation of the study is that the MRI evaluation was performed only in a subset of patients due to a lack of resources. However, we obtained paired T2∗ MRI of the liver and heart in 17/21 patients who completed the trial. We believe this limitation does not significantly impact the conclusions of the study.

In conclusion, this study has demonstrated that triple combination therapy with deferoxamine, deferasirox, and deferiprone is more efficacious in reducing the overall iron burden measured as serum ferritin and myocardial iron content in patients with transfusion-dependent β-thalassaemia with very high iron overload. Deferiprone has the disturbing side effect of reversible but severe arthropathy, which might limit the use of triple combination therapy, at least in some ethnicities. If tolerable, our findings support the use of the combination of deferoxamine, deferasirox, and deferiprone to chelate iron in patients of β-thalassaemia with very high iron overload.

## Contributors

AP conceived the study. AP, CP, RS, SW and SM contributed to the study design. AP, SW, CP, MNW, and SM contributed to the subject enrolment. AP, SW, CP, MNW, RR, RS, SW and SM contributed to the data collection. AP, SW, and SM performed data analysis. AP, SW and SM had directly accessed and verified the underlying data reported in the manuscript. AP and SM drafted the manuscript and AP, CP, MNW, SW, RR, RS, SW, and SM finalised the manuscript. All authors approved the final manuscript.

## Data sharing statement

Data will be available on a reasonable request to the corresponding author.

## Declaration of interests

We declare no competing interests.

## Appendix A Supplementary data

Protocol_Triple Therapy
