# Supplementary material for: Deferoxamine, deferasirox, and deferiprone triple iron chelator combination therapy for transfusion-dependent β-thalassaemia with very high iron overload: a randomised clinical trial
Source: Lancet Reg Health Southeast Asia. 2024 Oct 15;30:100495. doi: 10.1016/j.lansea.2024.100495 (PMC11718414; doi:10.1016/j.lansea.2024.100495)
Supplement: Supplementary Tables [file mmc2.pdf]

Supplementary Tables

Manuscript title: Deferoxamine, deferasirox, and deferiprone triple iron chelator combination therapy for transfusion-dependent β-thalassaemia with very high iron overload: a randomised clinical trial

Supplementary Table 1 – Exact doses of medication received by trial participants

Study Number	***Treatment arm***	***Gender***	***Age (years)***	***Weight (kg)***	***Deferoxamine dose (mg/od)***	***Deferasirox dose (mg/od)***	***Deferiprone dose (mg/tds)***
1	Intervention	Male	31	69	2000	2400	1500
2	Control	Male	36	60	1500	2400	-
3	Intervention	Female	31	69	1500	2400	1500
4	Intervention	Male	31	52	2000	2000	1250
5	Intervention	Female	32	45	2000	1800	1000
6	Intervention	Male	28	42	2000	1700	1000
7	Control	Male	25	45	1500	1800	-
8	Intervention	Male	28	59	2000	2200	1250
9	Intervention	Female	26	45	2000	1800	1000
10	Control	Female	28	28	1500	1200	-
11	Control	Male	25	50	2500	2000	-
12	Intervention	Male	21	53	2000	2000	1250
13	Intervention	Female	28	46	1500	1800	1000
14	Intervention	Female	15	35	1500	1400	750
15	Intervention	Female	26	70	2500	2400	1750
16	Control	Female	25	46	1500	1800	-
17	Control	Female	15	36	1500	1400	-
18	Intervention	Female	21	26	1000	1000	750
19	Control	Male	19	39	1500	1600	-
20	Control	Male	23	62	2000	2400	-
21	Intervention	Male	21	53	2000	2100	1250
22	Intervention	Female	19	59	2000	2400	1500
23	Intervention	Female	13	44	2000	1800	1000

Supplementary Table 2 – Description of trial defaulters

Study number	***Treatment arm***	***Duration of follow up (days)***	***Reason for defaulting***
6	Intervention	54	Died (Causes of death: Complications of Thalassaemia)
18	Intervention	120	Changed follow-up to a different thalassaemia centre

Supplementary Table 3 – Description of subjects deviated from the treatment protocol

Study number	***Treatment arm***	***Duration in the treatment protocol (days)***	***Reason for protocol deviation***
8	Intervention	157	Deferiprone stopped due to arthralgia
12	Intervention	32	Deferiprone stopped due to arthralgia
21	Intervention	81	Deferiprone stopped due to arthralgia
22	Intervention	7	Deferiprone stopped due to arthralgia
23	Intervention	69	Deferiprone stopped due to arthralgia

Supplementary Table 4 – Serum ferritin at 3 and 6 months compared to the baseline of the participants who completed the trial per-protocol

Number (%) showing reduction in serum ferritin compared to baseline	***Intervention arm (n=8)***	***Control arm (n=8)***	***OR (95% CI)***	**p-value**
At completion of 3 months	4 (50.0%)	6 (75%)	0.33 (0.04-2.76)	0.30
At completion of 6 months	8 (100%)	5 (62.5%)	10.8 (0.46-252)	0.05
Number (%) showing >500ng/mL reduction in serum ferritin compared to baseline	***Intervention arm (n=8)***	***Control arm (n=8)***	***OR (95% CI)***	
At completion of 3 months	4 (50.0%)	3 (37.5%)	1.66 (0.22-12.2)	0.61
At completion of 6 months	6 (75.0%)	4 (50.0%)	3.0 (0.36-24.9)	0.30
Change of serum ferritin from baseline (Mean±SD)	***Intervention arm (n=8)***	***Control arm (n=8)***	***Mean difference (95%CI)***	
At completion of 3 months	-310 (±997)	-192 (±639)	-117 (-1016 to 780)	0.78
At completion of 6 months	-1048 (±824)	82 (±1588)	-1131 (-2488 to 225)	0.09

Supplementary Table 5 – Change of liver iron content as measured by T2* MRI of the participants who completed the trial per-protocol[1]

Number (%) showing decrease in liver iron content compared to baseline	***Intervention arm (n=7)***	***Control arm (n=5)***	***OR (95% CI)***	**p-value**
Any decrease in liver iron content	4 (57.1%)	3 (60.0%)	0.88 (0.08 - 9.19)	0.92
More than 10% decrease in the liver iron content	4 (57.1%)	3 (60.0%)	0.88 (0.08 - 9.19)	0.92
Change of liver iron content from baseline	***Intervention arm (n=7)***	***Control arm (n=5)***	***Mean difference (95%CI)***	**p-value**
Mean(±SD) change of liver iron content (mg/g)	-0.40 (±3.7)	0.75 (±3.2)	-1.15 (-5.76 to 3.45)	0.58

[1] Only 12 participants (intervention-7; control-5) had pre- and post-treatment liver MRI due to limitation in facilities

Supplementary Table 6 – Change of cardiac T2* value of the participants who completed the trial per-protocol[2]

Number (%) showing increase in cardiac T2* compared to baseline	***Intervention arm (n=7)***	***Control arm (n=5)***	***OR (95% CI)***	**p-value**
Any increase in cardiac T2*	5 (71.4%)	1 (20.0%)	10.0 (0.64-154.3)	0.07
More than 10% increase in cardiac T2*	4 (57.1%)	1 (20.0%)	5.33 (0.37-75.7)	0.19
Change of cardiac T2* from baseline	***Intervention arm (n=7)***	***Control arm (n=5)***	*Mean difference (95%CI)*	**p-value**
Mean(±SD) change of cardiac T2* (ms)	8.20 (±11.2)	-3.00 (±8.24)	11.20 (-2.06 to 24.46)	0.08

[2] Only 12 participants (intervention-7; control-5) had pre- and post-treatment cardiac MRI due to limitation in facilities

Supplementary Table 7 – Details of the subjects who discontinued the trail due to the development of arthralgia

Study number	***Age (years)***	***Gender***	***Duration of treatment before arthralgia (days)***	***Joints involved***
8	28	Male	157	Knee joints
12	21	Male	32	Knee joints
21	21	Male	81	Knee joints
22	19	Female	7	Knee joints
23	13	Female	69	Ankle joints
